# Screening of Phytophagous and Xylophagous Insects Guts Microbiota Abilities to Degrade Lignocellulose in Bioreactor

**DOI:** 10.3389/fmicb.2018.02222

**Published:** 2018-10-03

**Authors:** Amandine Gales, Lucile Chatellard, Maider Abadie, Anaïs Bonnafous, Lucas Auer, Hélène Carrère, Jean-Jacques Godon, Guillermina Hernandez-Raquet, Claire Dumas

**Affiliations:** ^1^LBE, University of Montpellier, INRA, Narbonne, France; ^2^Laboratoire d’Ingénierie des Systèmes Biologiques et des Procédés, CNRS, INRA, INSA, Université de Toulouse, Toulouse, France

**Keywords:** fermentation, biomimetism, insect guts, volatile fatty acids, microbial diversity

## Abstract

Microbial consortia producing specific enzymatic cocktails are present in the gut of phytophagous and xylophagous insects; they are known to be the most efficient ecosystems to degrade lignocellulose. Here, the ability of these consortia to degrade *ex vivo* lignocellulosic biomass in anaerobic bioreactors was characterized in term of bioprocess performances, enzymatic activities and bacterial community structure. In a preliminary screening, guts of *Ergates faber* (beetle), *Potosia cuprea* (chafer), *Gromphadorrhina portentosa* (cockroach), *Locusta migratoria* (locust), and *Gryllus bimaculatus* (cricket) were inoculated in anaerobic batch reactors, in presence of grounded wheat straw at neutral pH. A short duration fermentation of less than 8 days was observed and was related to a drop of pH from 7 to below 4.5, leading to an interruption of gas and metabolites production. Consistently, a maximum of 180 mg_eq.COD_ of metabolites accumulated in the medium, which was related to a low degradation of the lignocellulosic biomass, with a maximum of 5 and 2.2% observed for chafer and locust gut consortia. The initial cell-bound and extracellular enzyme activities, i.e., xylanase and β-endoglucanase, were similar to values observed in the literature. Wheat straw fermentation in bioreactors leads to an increase of cell-bounded enzyme activities, with an increase of 145% for cockroach xylanase activity. Bacterial community structures were insect dependent and mainly composed of Clostridia, Bacteroidia and Gammaproteobacteria. Improvement of lignocellulose biodegradation was operated in successive batch mode at pH 8 using the most interesting consortia, i.e., locust, cockroaches and chafer gut consortia. In these conditions, lignocellulose degradation increased significantly: 8.4, 10.5, and 21.0% of the initial COD were degraded for chafer, cockroaches and locusts, respectively in 15 days. Consistently, xylanase activity tripled for the three consortia, attesting the improvement of the process. Bacteroidia was the major bacterial class represented in the bacterial community for all consortia, followed by Clostridia and Gammaproteobacteria classes. This work demonstrates the possibility to maintain apart of insect gut biological activity *ex vivo* and shows that lignocellulose biodegradation can be improved by using a biomimetic approach. These results bring new insights for the optimization of lignocellulose degradation in bioreactors.

## Introduction

With the depletion of fossil fuel reserves, it is nowadays important to find other ways to produce energy from renewable resources, and using environmental-friendly processes. The production of energy and chemicals by the enzymatic and/or microbial deconstruction of lignocellulosic biomass appears to be very attractive as lignocellulose is the most abundant renewable source of polysaccharides on earth and it is not in competition with food production (Carriquiry et al., 2011).

Lignocellulose is the principal structural component of plant cell wall which is composed of three major components: lignin, hemicelluloses, and cellulose (Menon and Rao, 2012). It is found in the most of industrial and agricultural wastes. Because of its complex structure and composition, it is for plant a natural barrier against biological attack (Fürstenberg-Hägg et al., 2013). However, this property is also an issue in the context of the biorefinery. During anaerobic digestion, the conversion efficiency of raw lignocellulosic biomass hardly reaches 30% (Sawatdeenarunat et al., 2015). To increase the biodegradability of lignocellulose and its conversion into bioenergy, pretreatments that are often expensive and not environmentally friendly need to be applied (Sun et al., 2016).

In Nature, different lignocellulose degradation processes exist that rely on combined action of a large number of microorganisms that produce lignocellulolytic enzymes, combined with other biotic and abiotic agents. These include the microbiome presents in the digestive tract of herbivorous and phytophagous organisms (Godon et al., 2013). Among them, phytophagous and xylophagous insects have been identified as one of the most effective lignocellulose digesters (Rizzi et al., 2013). Higher-termites are able to recycle 65–99% of the ingested cellulose and hemicelluloses respectively, whereas some scarabs can degrade up to 65% of the fibers present in their diet (Cazemier et al., 1997; Ohkuma, 2003). Since few decades, efforts are focused on the understanding of lignocellulose degradation in insect digestive tracts in order to use this knowledge to improve biomass conversion efficiency in bioreactors (Brune, 1998; Huang et al., 2010).

The gut of insects is divided into three parts, namely foregut, midgut and hindgut (Watanabe and Tokuda, 2010). The foregut is the place where mechanical treatments occur in order to reduce food particle size to less than 50 μm (Bayané and Guiot, 2011). The midgut has been identified as mainly anaerobic and alkaline with a pH close to 9–10, as usually used for alkaline chemical pretreatments, which are performed as an upstream process to delignify, expand fibers and increase biomass porosity and surface area (Watanabe and Tokuda, 2010; Monlau et al., 2015). Biological treatment, i.e., endogenous alkaline-tolerant enzymes such as cellulases (Shi et al., 2010; Willis et al., 2010) is associated with the chemical treatment to increase the lignocellulose hydrolysis. Finally, the hindgut is the compartment where symbiotic microorganisms are hosted and achieve the degradation of the remaining biomass (Watanabe and Tokuda, 2010). Indeed, working on lower termites, Breznak and Brune (1994) have shown that the bacterial flora is essential to biomass conversion and to the survival of insects.

The bacterial microbiota represents nearly a quarter of the insect weight and is mostly composed of protozoans and fermentative bacteria mainly belonging to four orders, i.e., Lactobacillales, Clostridiales, Bacillales, and Cytophaga-Flavobacterium-Bacteroides phylum (Huang et al., 2010). They ferment polysaccharides into acetate, carbon dioxide and hydrogen, using similar metabolic pathways as observed for dark fermentation as described by Guo et al. (2010).

To date, most of the research has been focused on the exploitation and cloning of endogenous or exogenous lignocellulolytic enzymes found in insects gut (Willis et al., 2010). Used in upstream pretreatment, these enzymes hydrolyse the raw biomass and release simple and fermentable sugars that will then be converted into valuable product. Very few articles had implemented the entire gut microbiomes in bioreactors and analyzed the degradation of lignocellulosic substrates (Hamdi et al., 1992; Auer et al., 2017). The advantage of using the whole microbiome resides in the robustness of the microbiome upon process variation such as change in substrate or operating conditions. Indeed, conversely to enzymes cocktail, entire gut microbiomes express a larger range of enzymes activities that are for interest in lignocellulose degradation.

In the present work, we examined in batch bioreactors the degradation activity of microbiomes from five xylophagous or phytophagous insects belonging to five different families in bioreactors. Our approach proposes to directly inoculate the whole microbiome of insects guts in anaerobic bioreactors using wheat straw as carbon source. The degradation of wheat straw was estimated in batch and successive batch conditions, without and with controlled pH respectively. The batch mode was limited due to acidification caused by fermentation. The three most performant consortia were then implemented in successive batch reactors with a pH controlled at 8. Changes of microbial diversity and enzyme activities were assessed throughout the wheat straw degradation processes; the feasibility of *ex vivo* utilization of guts microbiome is discussed.

## Materials and Methods

### Selected Insects, Insect Gut Preparation, and Lignocellulosic Substrate

Five species of insects were selected based on prior knowledge on their phytophagous or xylophagous diet and the availability of species in France. Chafer: Coleoptera *Potosia Cuprea* (Coleoptera, Cetonnidae, larvae stage) and Cockroach: Madagascar giant hissing cockroach *Gromphadorrhina portentosa* (Blattodea, adult and larvae stage L2–L3) were kindly provided by the Engineering School of Purpan (France). They were maintained in their natural environment and fed with wheat straw (variety Koréli). Beetle: *Ergates faber* (Coleoptera, Cerambycidae, larvae stage) were harvested in Aude department (France) inside dead pine trees and were also kept in their natural environment before use. Larvae of the Orthoptera, Locust and Cricket: adults of *Locusta migratoria* (Orthoptera, Acrididae) and crickets *Gryllus bimaculatus* respectively (Orthoptera, Gryllidae) were supplied by pet shops (Entomos, Coudekerque-Branche, and Tridome, Narbonne, France).

All insects were first anesthetized under CO_2_ flow and their bodies were surface sterilized with 70% ethanol. Gut of coleopterans, orthopterans and adult cockroaches were dissected in ultra-pure water in order to uptake guts. All guts were weighed and grounded with a blender in a minimum medium at pH 7 [in g⋅L^-1^: K_2_HPO_4_(0.45), KH_2_PO_4_ (0.45), NH_4_Cl (7.0), NaCl (0.9), MgCl_2_.6H_2_O (0.16), CaCl_2_.2H_2_O (0.09); in mg/l: Biotin (0.002), p-aminobenzoate (0.010), Thiamine (0.011), Pantothenate (0.005), Pyridoxamine (0.072), B12 Vitamin (0.020), Nicotinate (0.020), H_3_BO_3_ (0.0003), FeSO_4_.7H_2_O (0.0011), CoCl_2_.6H_2_O (0.00019), MnCl_2_.4H_2_O (0.00005), ZnCl_2_ (0.000042), NiCl_2_.6H_2_O (0.000024), NaMoO_4_.2H_2_O (0.000018), CuCl_2_.2H_2_O (0.000002)]. The lignocellulosic substrate used for the anaerobic cultivation was the same wheat straw as the one used for insect feeding (variety Koréli), ground at 150 μm with an impact mill (Ultrapez UPZ) and sterilized for 20 min at 121.1°C. Elementar analysis of wheat straw revealed a raw chemical formula equivalent to theoretical 1.354 gCOD⋅g^-1^.

### Batch Reactors

Three replicates of batch tests were analyzed in 120 mL penicillin flasks with 2 g of wheat straw (94% of total solids-TS) and 50 mL of insect gut suspension at 50 g⋅L^-1^(named wheat straw reactors WSR), resulting to a substrate to inoculum mass ratio of 1.25. A fourth flask was prepared without straw as a control in order to follow the metabolite production due to the degradation of residual gut tissues (named blank reactors: BR).

WSR and BR were operated under strict anaerobic conditions by sealing the flasks with rubber septa and flushing them with nitrogen for 10 min. Flasks were incubated for 15 days at 30°C with shaking at 150 rpm in an INNOVA43 incubator. During the incubation period, metabolites production (biogas and volatile fatty acid-VFA) was monitored at regular intervals.

### Successive Batch Reactors

To demonstrate the lignocellulose biodegradation ability of insect gut derived microbiomes, successive batch reactors (three successive batch named WS-SBR-1, WS-SBR-2, and WS-SBR3) were inoculated with the three selected consortia: cockroach, locust and chafer microbiomes. The successive batch reactor tests were conducted in successive batch modes. The initial volume was 400 mL of medium (described above) containing wheat straw (20 g⋅L^-1^). To standardize the number of bacteria per inoculum, the amount of 16S copies quantified by qPCR was used and the estimated number of bacteria was made with an average of 3.6 copies per genome (Sun et al., 2013). The substrate to inoculum mass ratio was then 7.47 for chafer, 2.5 for cockroach and 0.53 for locust. Bioreactors were maintained under anaerobic conditions at 30°C, under agitation at 300 rpm. The pH was monitored and controlled manually to 8 by addition of NaOH 1M once a day if needed. Throughout the incubation time, liquid samples were withdrawn regularly to analyze the production of soluble metabolites, enzymatic activities and microbial diversity. For diversity analysis, samples were immediately frozen at 80°C. After sampling, the working volume was completed to 400 mL for each reactor with 20 g⋅L^-1^ of wheat straw suspension in the minimum medium, which correspond to around 130–200 mL of medium depending the insect guts microbiomes considered.

### Substrate Characterization, Fermentation Products (Organic Acids), and Gas Analysis, Determination of Substrate Degradation

The wheat straw was composed of 94.3 ± 0.8 g of total solids per 100 g of wheat straw and 89.5 ± 0.3 g of volatile solids per 100 g of wheat straw The polysaccharide composition was determined by HPLC after acid hydrolysis (Monlau et al., 2012) and contained 18.7 ± 1.7% glucose; 16.5 ± 0.6% of xylose, 1.9 ± 0.0% of arabinose. For 100 g of wheat straw, these sugars represent 19.94 gCOD of glucose, 17.6 gCOD of xylose, and 2.03 gCOD of arabinose i.e., a total of 39.6 g of COD originating from sugars potentially hydrolysable and fermentable from wheat straw polymers.

Liquid samples were centrifuged and filtered at 2.7 μm (Whatman GF/D glass microfiber filters). The pH was measured immediately after sampling. Volatile fatty acids (VFA) from C2 (acetate) to C6 (caproate) were quantified with a gas chromatograph (Perkin Clarus 580) as described elsewhere (Motte et al., 2013).

Gas production was determined by pressure measurement. The composition of produced gas was analyzed by gas chromatography as presented by Motte et al. (2015).

The degradation of total COD reveals the degradation activity. As the only substrate fed into the reactors is wheat straw (after subtracting the blank reactors production that counts for insect guts tissues degradation), the COD degradation can be related to lignocellulose degradation.

The proportion of wheat straw degraded (in COD) during each experiment was evaluated as presented in Equation (1), with the assumption that the degradation of gut tissues produced the same amount of COD in both wheat straw and blank reactors.

The quantity of metabolites produced in BR also in eq.COD (difference between final [VFA+Gas]^Fin.^_BR_ and initial quantity [VFA]^Init.^_BR_) was subtracted to the quantity of metabolites produced in WSR in eq.COD (difference between final [VFA+Gas]^Fin.^_WSR_ and initial quantity [VFA]^Init.^_WSR_). This difference was then divided by the COD of wheat straw present in reactor (COD^init.^_WS_). The quantity of gas at the beginning of the reaction was equal to zero, that is why no gas appears for initial time in Equation (1).

$$\frac{{\left[VFA+Gas\right]}_{WSR}^{Fin.}-{\left[VFA\right]}_{WSR}^{Init.}-{\left[VFA+Gas\right]}_{BR}^{Fin.}-{\left[VFA\right]}_{BR}^{Init.}}{\left[{COD}_{WS}\right]}$$

### Total DNA Extraction

Total DNA was extracted from 1.5 mL samples according to the protocol of Rochex et al. (2008) including a heat-treatment step. Briefly, for cell lysis, samples were incubated with 5% N-lauroylsarcosine 0.1 M phosphate buffer (pH 8.0) at 70°C for 1 h and were shaken with 0.1 mm diameter silica beads (Sigma) for 10 min in a Vibro shaker (Retsch) at maximum speed. After adding polyvinylpolypyrrolidone, the tubes were vortexed and centrifuged. After recovering the supernatant, the pellet was washed with TENP (50 mM Tris [pH 8], 20 mM EDTA [pH 8], 100 mM NaCl, 1% polyvinylpolypyrrolidone) and centrifuged. Nucleic acids were precipitated by adding isopropanol (v/v) for 10 min at room temperature and then centrifuged. Pellets were incubated with RNase. Total DNA was finally purified using a QIAamp DNA minikit (Qiagen, Hilden, Germany) and suspended in water.

### Microbial Sequencing and Statistical Analyses

The highly variable V3–V4 region of the 16S rRNA gene was amplified from genomic DNA samples over 30 amplification cycles at an annealing temperature of 65°C, with the forward primer and the reverse primer with their respective linkers. The resulting products were purified, standardized to equimolar quantities and loaded on a MiSeq Illumina cartridge for sequencing of paired 460 bp reads following manufacturer’s instructions. Library preparation and sequencing were performed at the Genotoul Genome and Transcriptome Core Facility of Toulouse, France (get.genotoul.fr).

Sequencing data analysis was performed as previously described by Lazuka et al. (2015). Operational Taxonomic Units (OTU; 97% similarity) were defined using Mothur default parameters. OTUs presenting less than 10 sequences across all samples were removed. Samples were normalized to 15,000 high quality sequences. Sequences are available at this link: https://doi.org/10.15454/E6YRKX.

Community diversity was estimated using the Shannon diversity index. A R script was used to perform a hierarchical clustering using hclust function from the vegan R-package. Dissimilarities inputs for clustering were obtained using the function vegdist based on the dissimilarity Bray-Curtis indices.

Principal coordinate analysis (PCoA) was conducted to determine microbial community differences across insects classified by type of reactors. A Hellinger transformation had previously been carried out on the sequencing data using the vegan package and PCoA were conducted on the R-package Mixomics given the Hellinger distance matrix.

### Enzymatic Activities

Xylanase and endoglucanase (CMCase) activities were analyzed at the beginning and the end of the experiment. The enzymatic activity measurements were performed as described previously by Lazuka et al. (2015). The protocol enables to compare the analysis of free and cell-bound activities present in the supernatant and in the pellet respectively.

## Results and Discussion

### Screening of Insect Gut Microbiome Potential to Convert Lignocellulose in Anaerobic Batch Reactor

#### Characterization of Wheat Straw Bioconversion

The microbial potential of insect gut s microbiomes to degrade lignocellulose was evaluated by inoculating them in batch bioreactors with wheat straw as substrate under anaerobic conditions. The fermentation products, i.e., VFA and gasses, were quantified to compare the insect microbiota potential to convert wheat straw.

The fermentation was quite short for all insects in reactors since maxima of biogas and VFA produced were respectively reached before 100 h (4 days) and 200 h (8 days) of fermentation, (not shown). The pH of the broth varied from 7 (initial value) to 4.5 (final value). At such a low pH, hydrolysis and fermentation reactions are known to be blocked, which might explain the fermentation stop after several days (Veeken et al., 2000).

**FIGURE 1:**
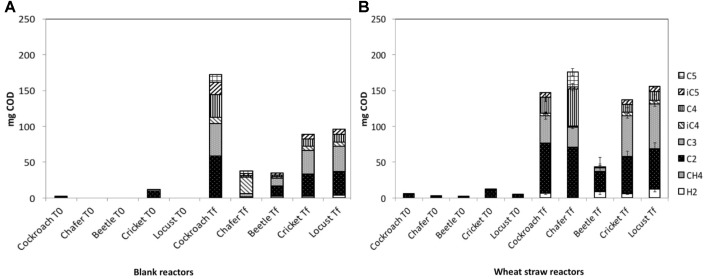
Production and repartition of volatile fatty acids (VFA) and gasses in mg_eq.COD_ observed in **(A)** blank reactors -BR- and **(B)** wheat straw reactors –WSR. Measured VFA are acetate (C2), propionate (C3), butyrate and its isomer (C4 and iC4), and valerate and its isomer (C5 and iC5). Measured gasses are hydrogen (H_2_) and methane (CH_4_). WSR reactors were fed with wheat straw and BR contained insect gut without wheat straw.

The volatile fatty acids (VFA) quantities already found in the medium before the fermentation process (T0), and the VFA and gas that accumulated at the end of the experiment (Tf) for both Blank Reactors (BR) and Wheat Straw Reactors (WSR) are shown in **Figure 1**. Quantities are expressed as chemical oxygen demand equivalent (mg_eq.COD_) to then be able to compare wheat straw degradation in function of introduced insect guts. The analysis of initial metabolites present in BR (corresponding to the quantity presents in the guts) and in WSR showed a nearly absence of organic acids except for crickets and cockroach guts microbiomes with acetate as the main product (10.9, 5.8 mg_eq.COD_ for WSR and 2.8, 9.5 mg_eq.COD_ for BR, respectively). Before the fermentation process, acetate was present in low quantity in all bioreactors containing wheat straw (WSR), with a maximum of 10.9 mg_eq.COD_ of acetate measured in bioreactors inoculated with cricket microbiomes. The presence of acetate in insect guts is consistent with the literature since it is generally the main final product of cellulose degradation in these biological systems (Zheng et al., 2012).

At the end of the experiment, the quantity of VFA and gas largely increased in all reactors, as an evidence of biological activity and biomass degradation. In BR, the presence of biodegradable compounds in insect guts and gut tissues can explain the production of metabolites in absence of wheat straw. In all cases, the biogas produced was composed of carbon dioxide and hydrogen (about 30 and 50%, respectively), with sometimes the presence of small quantities of methane. The production of H_2_ in insect guts has been identified as the result of cellulose degradation (Breznak and Brune, 1994). In the gut, other microorganisms transform then H_2_ into CH_4_, but this conversion is generally produced under strict environmental conditions and neutral pH. The drop of pH to 4.5 probably prevented methane production in the present study. Considering COD equivalents (eq. COD), H_2_ produced in WSR represented about 5% of total COD produced. Locust and beetle microbiota generated the highest quantity of H_2_ with respectively 12.4 and 8.6 mg_eq.COD_, followed by crickets (5.9 mg_eq.COD_). The lowest production of hydrogen was observed for chafers microbiota with 0.5 mg_eq.COD_, respectively. Concerning BR, the H_2_ produced ranged from 0.4 mg_eq.COD_ in cockroach reactors to 3.9 mg_eq.COD_ in locust reactors. As stated before, BR only contained gut tissues as substrate, biomass richer in protein than wheat straw. This difference of substrate resulted in a lower production of hydrogen in BR. It has been observed elsewhere that hindgut of crickets fed on rich protein diet generates 3.5 times less of H_2_ than hindgut of cricket fed on lignocellulosic biomass (Santo Domingo et al., 1998).

The production of VFA in WSR ranged from 150 to 180 mg_eq.COD_ for chafer, locust, cockroach and cricket microbiota, whereas only 50 mg_eq.COD_ were measured in reactors inoculated with beetles microbiota. VFA produced were essentially acetate with 28 and 70 mg_eq.COD_ measured in beetle and chafer bioreactors, respectively. Except for beetles, propionate was produced in similar quantity for all insects, ranging from 27 to 62 mg_eq.COD_ for chafer and locust, respectively. In control reactors (BR), the main VFAs produced were acetate, with 14–58 mg_eq.COD_ for beetles and cockroaches reactors respectively, and propionate with 11–45 mg_eq.COD_ for beetles and cockroaches reactors respectively. In chafer reactors, a large part of butyrate was produced corresponding to 23 mg_eq.COD_. In all reactors, the presence VFA such as butyrate, valerate and their isomers can be the result of the sudden stop of fermentation due to pH drop as mentioned above. Indeed, in insect gut, complex biomass such as cellulose is first hydrolyzed and converted into three or more carbon organic acids, which are further converted into acetate, hydrogen and finally methane (Breznak and Brune, 1994).

Considering the COD recovered only as metabolites enabled to estimate a net wheat straw degradation (that could be related to lignocellulose degradation) which is maximal for chafer (5%), followed by locust (2.2%), cricket (1%), and beetle (0.3%) microbiomes (**Table 1**). The corresponding value for cockroach microbiomes could not be determined since the quantity of COD produced in BR, containing only gut tissues, was higher than that measured in WSR. As previously mentioned, this low proportion of biomass degraded could result from the pH drop observed after few days of incubation, but also by the experimental conditions that were not optimized for gut microbiomes. A neutral pH was selected for this experiment, as it is a value usually used in anaerobic digestion and because is usually encountered in hindgut of most insects (Engel and Moran, 2013; Brune, 2014). However, lignocellulose degradation is higher in insect gut particularly when alkaline conditions prevail since high pH promotes the cleavage of lignin-carbohydrate complex (Engel and Moran, 2013; Brune, 2014).

**Table 1:** Biodegradation yield of wheat straw introduced in batch reactors (in % of eq.COD).

Insect guts microbiomes	Cockroach	Chafer	Beetle	Cricket	Locust
% Degradation of wheat straw (eq.COD)	/	5.0	0.3	1.0	2.2


The quantity of gut tissues used as reactors inocula could have negatively affected lignocellulose degradation. Indeed, the biodegradation of this organic biomass is easier than that of wheat straw. Thus, the lignocellulosic biomass would be attacked only after gut tissues were converted into metabolites. This assumption could be confirmed through the degradation of initial COD in BR since between 12 and 46% of gut COD were converted into metabolites (data not shown) whereas only a maximum of 5% for wheat straw was degraded.

Using a pH value close to neutral conditions but slightly alkaline as measured in insect guts (**Supplementary Table S1**) and implementing a pH control might improve lignocellulose degradation rate. Similarly, a methodology to reduce the amount of gut-tissue inoculated in the bioreactors has to be developed in order to select more performant microbial communities able to degrading lignocellulose.

#### Initial Enzymatic Activity in Insect Guts Microbiomes and Residual Enzymatic Activity Profile After Wheat Straw Fermentation

**FIGURE 2:**
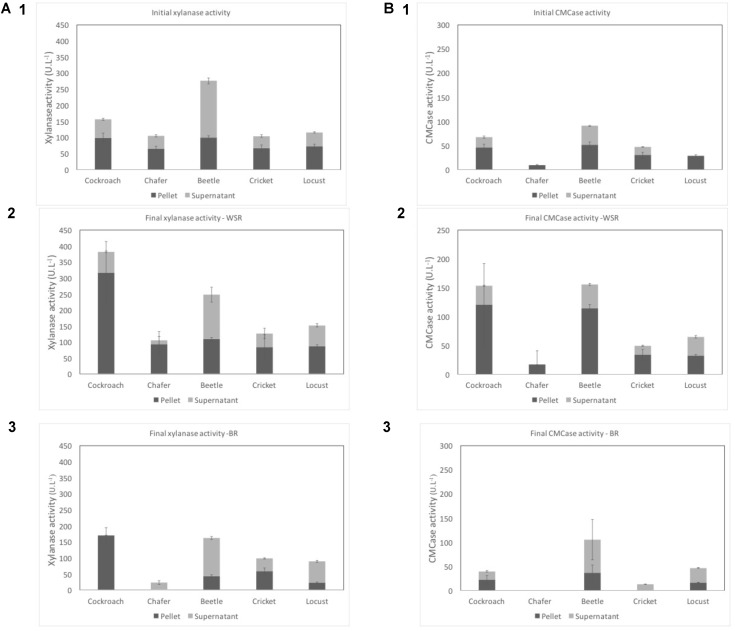
Xylanase **(A)** and CMCase **(B)** activities in U.L-1 before the fermentation **(1)** and after the process for WSR **(2)**, and for BR **(3)**. Dark gray represents cell-bound activities (pellet) and light gray the free enzymatic activities (supernatant). WSR reactors were fed with wheat straw and BR contained insect gut without wheat straw.

Both initial and final enzymatic activities, i.e., xylanase and endoglucanase (CMCase) activities, of the insect gut microbiota were measured at the beginning and at the end of incubation in bioreactors experiments (**Figure 2**). The applied protocol enabled to measure both extracellular and cell-bound enzyme activities, present in the supernatant and in the pellet, respectively.

Before the fermentation, chafer, cricket and locust exhibited similar xylanase activity close to 100 U⋅L^-1^ (a.1). The maximal xylanase activities after the fermentation period were observed for beetle (276 U⋅L^-1^) and cockroach (156 U⋅L^-1^). These last insect gut microbiomes, therefore, seemed to have a higher initial potential to degrade xylan. A similar xylanase activity value (200 U⋅L^-1^) was previously obtained by Lazuka et al. (2015) using rumen microbiomes as inoculum to degrade wheat straw in anaerobic bioreactors. However, Shi et al. (2010) observed a xylanase activity of 0.1 U⋅L^-1^ for woodborer insect guts. In this study, xylanase activity was performed on the juice content of the guts after dissection. In the present study, initial xylanase activity was mainly detected in the pellets meaning that the enzymes were preferentially cell-bound. One exception is noted for beetles with the maximum activity detected in the supernatant. Cell bounded hydrolytic enzymes have also been identified in rumen when this microbiomes was used as inoculum for wheat straw degradation in the study of Lazuka et al. (2015).

Regarding CMCase activity, its initial activity (**Figure 2B1**) was also higher in the pellet than in the supernatant for all insects. The highest global activity was observed for beetle and cockroach with 91 U⋅L^-1^ and 67 U⋅L^-1^, respectively and the lowest was measured in chafer reactors at 9 U⋅L^-1^. In their study, Lazuka et al. observed an initial CMCase activity close to 20 U⋅L^-1^. But higher endoglucanase activity was observed for the sugarcane borer *Diatraea saccharalis* larvae, with free enzymatic activity of 200–300 U⋅L^-1^ (Dantur et al., 2015). In this case, enzymatic activity was performed after selection of intestinal bacteria on cellulose as sole carbon source, which are thus supposed to exhibit higher endoglucanase activity that observed in the present study.

After the fermentation process, a global increase of enzymatic activities was observed, from 22 to 145% for xylanase and 5–128% for CMCase. The increase of activity was higher in the pellet with a maximum of 221% observed for xylanase activity in cockroach microbiome. At the opposite, the free enzyme activities, measured in the supernatant, globally decreased for chafer, beetle (xylanase) and cricket (CMCase).

Chafer microbiomes exhibit the lowest enzymatic activities both with and without wheat straw (94 and 12 U⋅L^-1^ for respectively pellet and supernatant activities and 17 U⋅L^-1^ for pellet CMCase activity, supernatant CMCase activity was too low to be detected).

In comparison with rumen microbiome systems, the xylanase activity measured after wheat straw degradation was higher than observed in the present study, with values reaching 800 U⋅L^-1^ (Lazuka et al., 2015). At the opposite, the same authors reported a CMCase activity of 40 U⋅L^-1^, similar to the value obtained here for chafer, but 2–6 times lower than the CMCase activity observed for the other insects.

The xylanase and CMCase activities of beetle gut microbiomes measured in the present study were quite the same at both initial and final stage. One hypothesis could be that beetle larvae were harvested in dead pine trunks (wood eater). Therefore, the difference between initial and final activity is lower since at initial stage a high potential to degrade xylan and cellulose was already detected.

Enzymatic activity, which represents a global degradation rate, was standardized by the 16S rRNA copies present in the sample which correspond to the witness of microbial biomass quantity. In this case, locust microbiomes exhibited the highest specific enzyme activities at initial and final stage whereas chafer guts microbiota still had the lowest specific enzyme activities (data not shown). It is striking to note that the specific enzyme activity decreased considerably between the beginning and the end of the experiment. Indeed, even if the global activity was quite the same, the bacterial growth resulted in an increase of 16S copies. At the end of the experiment, the global activity was similar to that of the beginning, but when considering the growth of bacteria, the specific activity was considerably reduced. Here again, the drastic drop of pH observed above can explain the decrease of enzymatic activity at the end of the process. Sampling during the fermentation period could have shown an increase of enzymatic activity during the exponential step of degradation.

Regarding specific enzymatic activity results, locust microbiota is an interesting candidate since it exhibits high xylanase and CMCase specific activities. Enrichment of such population could be interesting for developing lignocellulose degradation bioprocesses.

#### Distribution and Bacterial Community Structures Across Samples Before and After Wheat Straw Fermentation in Batch Experiments

**FIGURE 3:**
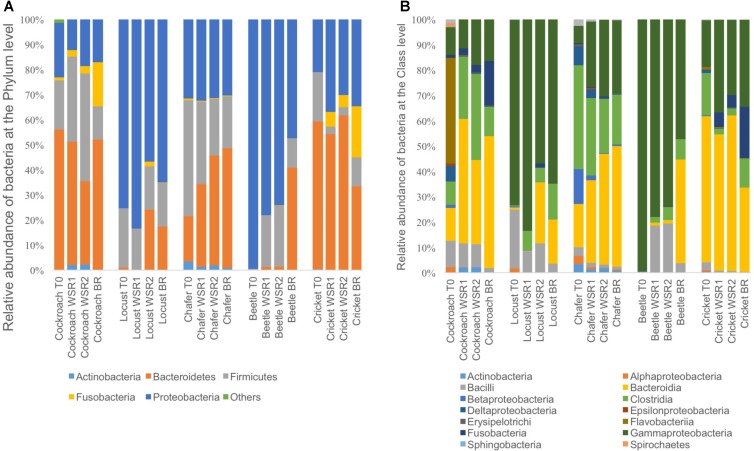
Relative abundance of bacteria phylum **(A)** and bacteria class **(B)** identified from the Illumina Miseq sequencing data, within insect gut microbiomes before (T0) and at the end of cultures in batch anaerobic reactors: duplicate WSR1 and WSR2 reactors were fed with wheat straw and BR contained insect gut without wheat straw.

Microbiomes from the insect gut s introduced in the bioreactors were sequenced using the MiSeq Illumina technology. The taxonomic assignment showed that 5 phyla were represented (**Figure 3**) including Actinobacteria, Firmicutes, Fusobateria, Bacteroidetes and Proteobacteria.

**FIGURE 4:**
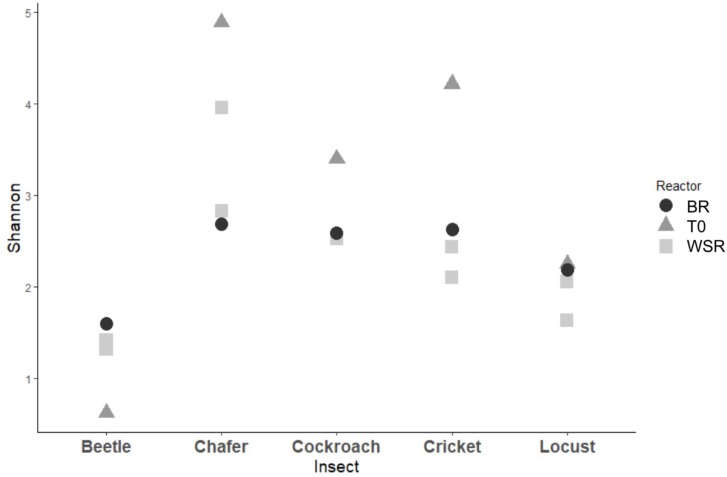
Diversity among bacterial community present within insect gut microbiomes before (T0) and after (WSR and BR) cultures in batch mode anaerobic reactors. Shannon diversity index evaluated at 97% similarity.

The 3 dominant phyla, i.e., Bacteroidetes, Firmicutes and Proteobacteria, were identified in 4 of 5 insect gut microbiomes, which is consistent with data obtained in another study across 21 taxonomic orders of insects (Yun et al., 2014). Actinobacteria and Fusobacteria phyla were poorly represented and Actinobacteria were found only in cockroach and chafer microbiota. Shannon diversity index was used to estimate the diversity of the community (**Figure 4**). The index range from 0, low diversity, to 5, high diversity. The lowest species diversity, Shannon index = 0.62, was harbored by the wild-caught beetle microbiomes, composed over 99% of Proteobacteria (Enterobacteriaceae). At the opposite, the soil-dwelling chafer larvae belonging to the same taxonomic insect order exhibited greater Shannon index (4.9) and its microbiome was dominated by the Firmicutes phylum (46%). The difference of Shannon diversity index observed can be explained by the difference of diet between the two insect species as observed in previous works (Kim et al., 2017; Su et al., 2017). In relation to their restricted habitat, beetles used in this study fed only on deciduous trees whereas chafer larvae fed with more diverse food composed of decaying plant material and plant roots. The digestion of more diverse food would be expected to involve more diverse bacteria species as proposed by Yun et al. (2014).

Cockroaches gut microbiota exhibited 56% of Bacteroidetes with an initial content close to 42% of *Blattabacterium* sp. (*Flavobacteria*). This intracellular mutualist, obligate endosymbiont, is shared in nearly all cockroaches host gut communities (Sabree and Moran, 2014). Regarding bacteria class taxonomic affiliation, sequences assigned to the Bacteroidia and Clostridia were predominant in crickets and chafers microbiota samples, with a cumulated abundance of 74 and 58% respectively. Proteobacteria phylum was also dominant in locust microbiota samples (75%) with 58% of Gammaproteobacteria (Enterobacteriaceae). The class Bacilli, mainly represented by the Lactobacillaceae family, was initially found in all gut microbiota, except in the beetle sample.

At the end of the fermentation, analysis of bacterial diversity revealed a decrease of the Shannon diversity index in bioreactor communities, except for beetles (**Figure 4**). The low diversity at the beginning of the experiment for beetles could be explained by the presence of one dominant OTU which represented more than 79% of the sequences. The fermentation process also resulted in changes of gut microbial community composition.

The 3 dominant phyla present at initial stage were also harbored with the predominance of the Clostridia, Bacteroidia and Gammaproteobacteria classes for all the insect microbiomes, in all conditions (WSR and BR) but with different the relative abundance. It has been shown that some bacteria belonging to the Clostridia phylum can produce cellulosomes, cell-bound cellulase complex that is known to be highly efficient in cellulose fiber degradation (Christopherson and Suen, 2013). The high level of cell-bound enzyme activity measured in the previous section (Initial enzymatic activity in Insect Guts Microbiomes and Residual Enzyme Activity Profile After Wheat Straw Fermentation) might be provided by this type of bacteria.

Compared to the initial microbial community, cockroach microbiomes lost the obligate endosymbiont *Blattabacterium* sp identified before; at the opposite, new bacterial species affiliated to the Bacteroidia class were identified in these microbiomes. Interestingly, Enterobacteriaceae family was represented at more than 10% in all samples and was always dominant in locust and beetles gut communities. Fermentative beetle communities harbored also an increase of the Bacilli class compared to the initial community, with more than 7% of Lactobacillaceae (data not shown).

**FIGURE 5:**
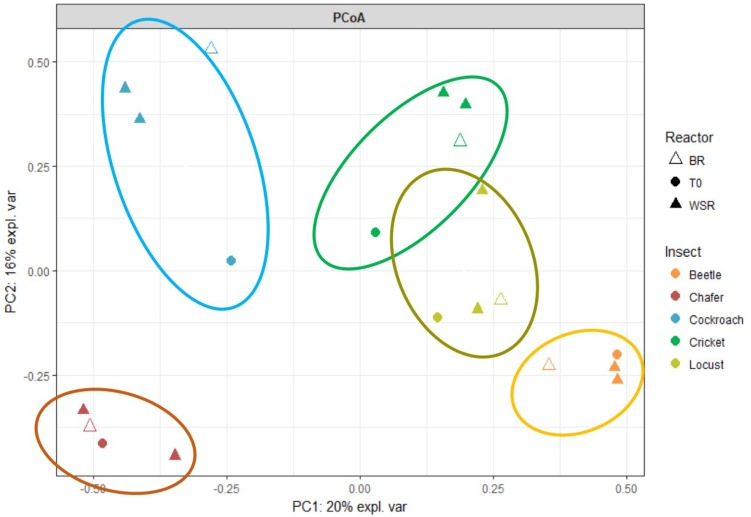
Principal coordinate analysis (PCoA) plot showing similarity relationships among bacterial community sampled from three groups of insect bioreactors (36% of the total variance explained).

The variability between the different bacterial community structures was explored using a principal coordinate analysis (PCoA) in **Figure 5**.

Microbiomes belonging to the same insect species form tight clusters, different from one insect species to another. However, replicates tended to be closely related to their bacterial community evolution (except locusts and chafers).

Change of bacterial composition of the microbiome from the initial inoculum to this composition at the end of the fermentation in batch conditions showed that the differences between insect species were maintained in the bacterial communities after the incubation period (15 days).

No large differences between WSR and BR were observed at the end of the fermentation (except for beetle). This observation could be related to the previously mentioned assumption that the fermentation had mainly occurred through the degradation of guts tissues. Thus, the WSR and BR communities were driven by the same factors.

This first experiment showed that it was possible to implement the whole microbiome of insect gut in usual anaerobic batch bioreactors. Even if the native insect gut microbiomes were quite different in terms of microbial diversity structure and potential, similar biological behaviors as observed *in vivo* have been highlighted such as similar bacteria species and enzyme activity level. However, technical issues were observed, making difficult to attest the efficiency of the wheat straw degradation process.

Further experiments were thus performed in successive batch mode using the three insects, i.e., cockroaches, chafers, and locusts. The choice of the three insects was based on the performances during the batch experiments, the phylogenetic distance and that availability of the insects. In this case, pH was controlled at 8, a value corresponding to the foregut of the studied insects.

### Lignocellulose Degradation in Successive Batch Conditions and Evaluation of Microbial Diversity Evolution

#### Bioconversion and Biodegradation of Wheat Straw

Here, the pH was adjusted and controlled to 8 which was the value measured *in situ* in the three insects guts (**Supplementary Table S1**).

**FIGURE 6:**
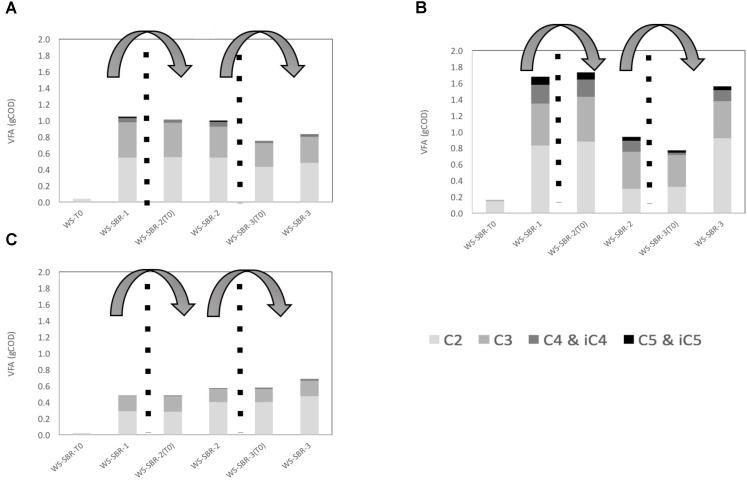
Production and repartition of volatile fatty acids (VFA) in successive batch reactors inoculated with **(A)** cockroach **(B)** locust and **(C)** chafer microbiome in mg_eq.COD_. Measured VFA are acetate (C2), propionate (C3), butyrate and its isomer (C4 and iC4), and valerate and its isomer (C5 and iC5).

The majority of COD from fermentation products consisted of VFA, the COD from gasses being negligible since the majority was CO_2_ (**Figure 6**). The VFA at the end of batches were mainly composed of acetate and propionate irrespective the insect microbiome. The quantity of VFA increased considerably between the beginning and the end of WS-SBR1, from 0 to around 0.5, 1 and 1.8 g_eq.COD_ for chafer, cockroach and locust respectively. The quantity of VFA was roughly maintained along the following batches without significant increase or decrease (except in cricket WS-SBR2).

Wheat straw biodegradation yield was estimated at the end the first two batches by subtracting the metabolites initially present in the medium and the end- products (metabolites and gasses) produced through insect guts degradation (control without wheat straw), to the total of metabolites and gasses measured (**Table 2**). After only one batch in alkaline conditions; WS-SBR-1, which lasted between 10 and 15 days, wheat straw was degraded in significant higher amount than observed during the screening experiment. Indeed, cockroach and chafer gut microbiomes enabled the degradation of 10.5 and 8.4% respectively, of wheat straw COD, whereas locusts gut microbial community could degrade 20.4% of raw wheat straw. Biological methane potential showed that only 54% of wheat straw is considered as degradable (data not shown), therefore the degradation yields measured in successive batch reactors are rather interesting. These experiments show that adapting environmental conditions could significantly increase the performances of guts microbial communities.

**Table 2:** Biodegradation yield of wheat straw introduced in sequential batch reactors (in % of eq.COD).

Insect guts microbiomes		Cockroach	Chafer	Locust
% Degradation	WS-SBR-1	10.5 ± 1.2	8.4 ± 1.5	21.0 ± 2.9
of wheat straw	WS-SBR-2	2.9 ± 0.6	7.8 ± 4.4	2.5 ± 1.9
(eq.COD)	WS-SBR-3	2.6 ± 1.5	2.1 and 0	/


During WS-SBR-2, the degradation yields decreased considerably. They dropped from 10.5 to 2.9% for cockroach WS-SBR-2, from 8.4 to 7.8% for chafer WS-SBR-2 and from 21 to 2.5% for locusts WS-SBR-2. For these degradation yields, it should be noted that around 3/4 of the reactor contained less biodegradable WS (from 16 to 40% depending the degradation rate obtained in WS-SBR-1), only 1/4 being represented by fresh WS (degradable at 54% as mentioned previously) making the global degradable fraction of WS diminishing around 30–45%. However, results show that the successive batch experiments performed here enabled to increase biodegradation during WS-SBR-1 but this ability was not maintained in the following cultivation cycles. The retention time used during this experiment might be too low for slow-growing microorganisms of interest that might have not grown during the process (Shi et al., 2017). Little information is available concerning implementation and taming of insect guts microbiomes as a whole in bioreactors. Some articles related to utilization of termite guts protein extracts (Rajarapu and Scharf, 2017) or microbiomes as a whole (Hamdi et al., 1992; Auer et al., 2017) but to our knowledge no information could be found concerning other type of insects implemented in bioreactors.

#### Enzymatic Activities Measured for the Three Selected Microbiota

**FIGURE 7:**
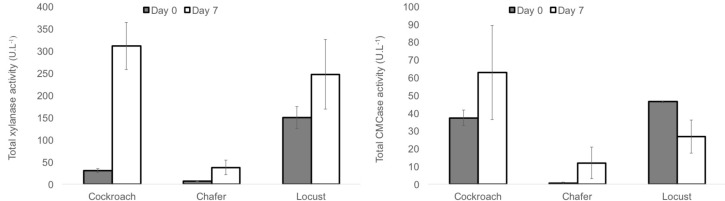
Total xylanase and CMCase activity measured for WS-SBR-1 at day 0 (dark gray) and day 7 (white).

The enzyme activity was measured during the fermentation. The total xylanase and CMCase activities at day 0 and day 7 (final point of exponential phase) are represented in **Figure 7** for the three insect guts microbiomes.

The xylanase activity had more than tripled (around 400 U⋅L^-1^) compared to that obtained in batch mode reactors (100–150 U⋅L^-1^) while the initial activities were quite the same and less than 50 U⋅L^-1^. These results might be explained by the conditions used for the successive batch reactors (monitoring and control of pH at 8) which appeared as more appropriate for these microbiomes. This global higher enzymatic activity was also observed through the degradation yield of wheat straw. Comparing the activity between insect shows that cockroach and locust guts microbiomes present the same enzymatic activities whereas chafer displayed significantly lower levels (around 50 U⋅L^-1^).

Concerning CMCase activity, it was quite low (under 100 U⋅L^-1^ for the three insects), the variations of activity between initial point and point 7 days are dissimilar for the three insects. Indeed the activity increased from around 40–60 U⋅L^-1^ for cockroach gut microbiomes. For chafer, it increased from quasi inexistant activity to around 10 U⋅L^-1^. Conversely, the activity decreased from 46 to 26 U⋅L^-1^ for locust guts microbiome.

#### Microbial Diversity

**FIGURE 8:**
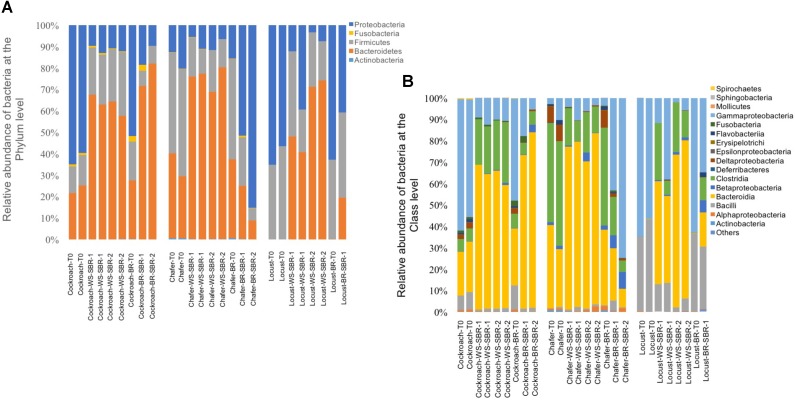
Relative abundance of bacteria phylum **(A)** and bacteria class **(B)** identified from the Illumina Miseq sequencing data, within insect gut microbiomes before (T0) and after cultures in successive batch reactors maintained at pH8: duplicate WS-SBR-1 and WS-SR-2 were fed with wheat straw and BR-SBR contained insect gut without wheat straw among two successive batch.

The fermentation under controlled pH conditions resulted in a change of gut microbiomes structure and diversity for the three insects in all reactors (**Figure 8**). The community structures of WSR shifted from microbiomes dominated by Bacteroidetes and Proteobacteria to Bacteroidetes and Firmicutes phyla. Among these phyla, Bacteroidia and Clostridia classes became preponderant except for the Chafer, where the Clostridia were always dominant. Proteobacteria phyla tended to be less abundant after fermentation of wheat straw (WSR-SBR reactors) and the relative abundance of Actinobacteria class decreased.

**FIGURE 9:**
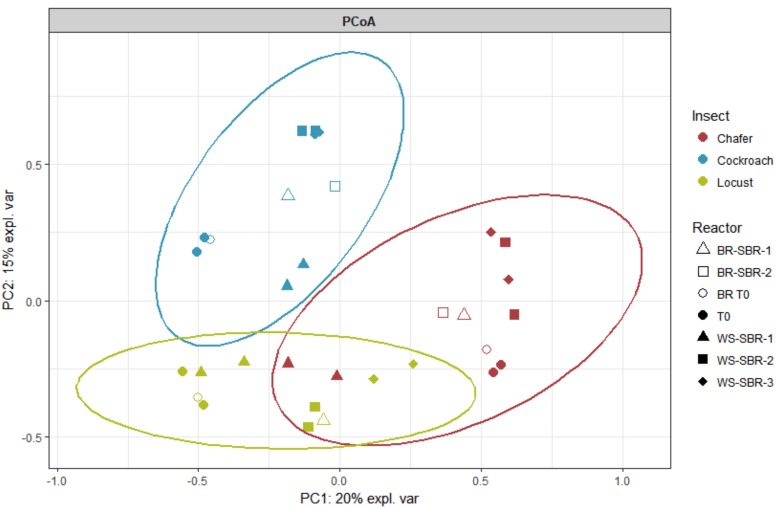
Principal coordinate analysis (PCoA) plot showing similarity relationships bacterial communities present within insect gut microbiomes before (T0) and at the end (WS-SBR and B-SBR) of cultures in successive batch reactors maintained at pH8. PCoA was conducted given the Hellinger distance matrix.

**FIGURE 10:**
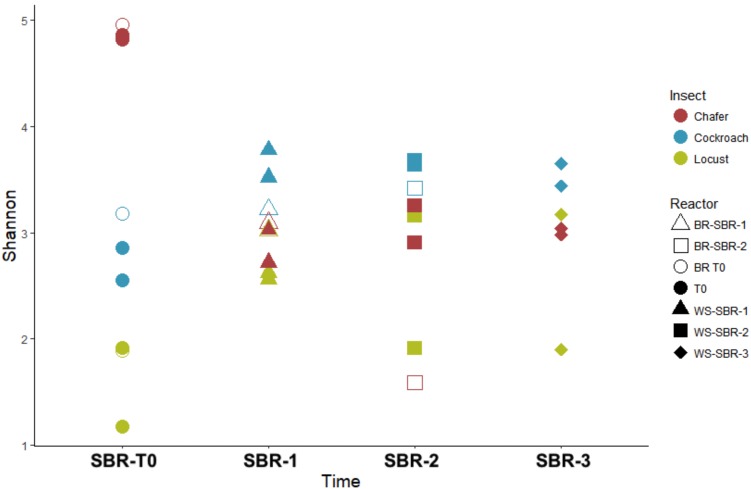
Diversity among bacterial communities present within insect gut microbiomes before (T0) and at the end of (WS-SBR-1, WS-SBR-2, and BR-SBR) cultures in successive batch reactors maintained at pH8. Shannon diversity index evaluated at 97% similarity.

As shown by the PCoA analysis (**Figure 9**), the structure of the community greatly shifted compared to the initial inoculum during the first batch and then, the compositions of the bacterial community tended to be stable after WSR-SBR-1. The major changes observed in alpha diversity occurred during the first batch of fermentation (**Figure 10**). The controlled conditions seemed, then, to minimize the bacterial community shifts of WSR for the 3 insects during the second batch. Moreover, theses results show that BR harbored modifications of community structures during first batch (BR-SBR-1) but remained distinct from WSR.

This suggests that the difference in composition and availability of substrate in BR and WSR (guts tissues and wheat straw respectively) also influence the shifts of the gut microbiomes. In agreement with these results, the **Figure 8** shows that chafer and locust microbiomes present in BR harbored more Proteobacteria than in WSR and more variable relative abundance of Firmicutes.

## Conclusion

Phytophagous and xylophagous insects are for sure the best living bioreactor model to degrade lignocellulosic substrate. This work showed for the first time a comparison of insects-guts microbiomes from different order as inocula for fermentation of lignocellulose. This comparison was done in term of enzymatic activity, microbial diversity, production of metabolites and degradation of wheat straw. This work also highlights the technical difficulty of these comparisons.

The possibility to use directly their whole microbiome in anaerobic batch reactors to ferment wheat straw was explored. Starting from neutral pH, cockroach and beetle microbiomes exhibited the highest global enzyme activity. The highest lignocellulose conversion was attributed to chaffer and locust with a maximum of 5% of lignocellulose converted into metabolites. The microbial diversity and enzymatic activities revealed to be very different depending the insect microbiome considered even if similar degradation potentials were obtained. By selecting environmental conditions closest to the insect gut, it was possible to improve the degradation of lignocellulose at a maximum of 20%. The control of pH allowed a good stability of fermentative bacterial community structure for reactors inoculated with chafer and cockroach microbiomes. Unfortunately, it was not possible to maintain a stable degradation of lignocellulose along the different batches. *Ex vivo* experiments strongly modify the structure of microbial communities. Works remain to maintain the biological function in reactors in relation to the bacterial community structure.

However, the high diversity of insect microbiome and the ability of these microbiomes to cumulate hydrogen and acetate *ex vivo* in bioreactors showed the high potential of this system for biotechnological application.

## Author Contributions

AG and LC are students in this project, did all bioreactors’ experiments, insects selection and dissection, data interpretation, and drafted the manuscript. LA and AG are students in this project and carried out the molecular diversity analysis. MA is a student in this project and did the enzymatic activity assays. AB is technician in this project and assisted LC and AG with the DNA extraction. J-JG, HC, CD, and GH-R participated in the experimental design and project conception, and critically revised the manuscript for important intellectual content. CD and GH-R are the main supervisors. CD is the project coordinator. All authors read and approved the final manuscript.

## Conflict of Interest Statement

The authors declare that the research was conducted in the absence of any commercial or financial relationships that could be construed as a potential conflict of interest.
