# Supplementary material for: Screening of Phytophagous and Xylophagous Insects Guts Microbiota Abilities to Degrade Lignocellulose in Bioreactor
Source: Front Microbiol. 2018 Oct 3;9:2222. doi: 10.3389/fmicb.2018.02222 (PMC6178917; doi:10.3389/fmicb.2018.02222)
Supplement: Supplementary file 1 [file Table_1.DOCX]

Table S1: pH along digestive tracts of insects

|  | Foregut | Midgut | Hindgut |
| --- | --- | --- | --- |
| Locust | 6 | 7 | 8 |
| Potosia | 7 | 9-10 | 8 |
| Gromphadorhina | 6 | 7 | 8 |
